# Regulation and activity of the phosphatase SHP2: SH2 domains, dephosphorylation activity, and beyond

**DOI:** 10.1042/BST20253102

**Published:** 2026-01-09

**Authors:** Catia L. Pierotti, Maja Köhn

**Affiliations:** 1 Department of Molecular Cell Biology, Institute for Cell Biology, University of Bonn, Bonn, 53115, Germany

**Keywords:** adaptor proteins, enzyme–substrate interactions, protein–protein interactions, protein tyrosine phosphatases, signaling, SH2 domains, SHP2

## Abstract

Src homology 2 (SH2) domain-containing phosphatase-2 (SHP2, *PTPN11*) is implicated in diseases such as cancer and RASopathies, where it is often mutated. It has gained strong attention due to promising new drug development strategies, with drug candidates currently in clinical trials. SHP2 is activated downstream of cell surface receptors to promote signaling pathways involved in cell growth and to inhibit immune cell activation. The phosphatase has two SH2 domains and a protein tyrosine phosphatase (PTP) domain, is post-translationally modified, and can function as an active phosphatase or as an adaptor/scaffold protein. It is subject to tight regulation in its cellular environment, for which novel insights have recently emerged. In this focused review, we first summarize the roles of the two SH2 domains and phosphorylation on the regulation of wildtype SHP2. We then describe new developments concerning catalytic and non-catalytic functions of SHP2, as well as recent progress in the understanding of SHP2 regulation, including it being subjected to SUMOylation, activated independently of cell surface receptors, and regulated by substrate phosphorylation. These new insights not only demonstrate the complexity of SHP2 regulation but also guide future studies, contributing important insights that could aid in targeting SHP2 in different disease contexts in the future.

## Introduction

Classical protein tyrosine phosphatases (PTPs) regulate intracellular signaling processes by removing the phosphate group from a phosphorylated tyrosine (pY), thereby counteracting protein tyrosine kinases that transfer the phosphate to the tyrosine. These processes are key to cellular life, as they control cell growth, division, differentiation, migration, and the decision between survival and death [1]. Src homology 2 (SH2) domain-containing phosphatase-2 (SHP2, gene name *PTPN11*) is an essential PTP that regulates cell signaling events controlling many of these important processes. SHP2 is a positive regulator of growth pathways, most prominently the mitogen-activated protein kinase (MAPK) pathway, downstream of cellular surface receptors that are stimulated by growth factors, cytokines, or other ligands [2–4]. As such, it is involved in promoting oncogenic signaling downstream of mutated receptor tyrosine kinases (RTKs), and it is itself also a proto-oncogene, as different mutations in SHP2 lead to activation of the phosphatase independently of receptor activation [3,4]. SHP2 also plays a role in adaptive drug resistance, whereby the combined pharmacological targeting of RTK signaling and SHP2 can prevent adaptive resistance in certain tumors [5–7].

Additionally, in T lymphocytes, SHP2 not only acts downstream of the T cell receptor to promote T cell activation but can also act directly downstream of the programmed cell death protein 1 (PD-1) receptor to inhibit T cell activation. Cancer cells can express PD-L1, a ligand that stimulates PD-1, to evade the immune response through this PD-1/SHP2 mediated signaling [8–10]. Therefore, this is an important process targeted by drugs in cancer immunotherapy, placing SHP2 at a key interference point in cancer immune evasion [11]. However, the importance of SHP2 in inhibitory PD-1 signaling has been questioned by several studies, which showed that *Ptpn11* deletion in mice T lymphocytes has surprisingly no effect on PD-1 function [12–14]. This conflicting evidence could be potentially due to SHP1, or another tyrosine phosphatase, compensating for the loss of SHP2 [13,15,16], or due to differences in PD-1 signaling between mice and humans [17]. Furthermore, in the innate immune response, SHP2 acts downstream of Toll-like receptors (TLRs) to negatively regulate interferon β production [18], and it suppresses TLR-triggered inflammatory responses downstream of major histocompatibility complex class I molecules [19]. SHP2 also localizes to mitochondria, where it maintains mitochondrial homeostasis and alleviates NOD-like receptor family pyrin domain containing 3 (NLRP3) inflammasome activation [20].

In addition to these roles, SHP2 is a key protein in RASopathies, which are a group of developmental syndromes caused by germline mutations in genes belonging to the extracellular signal-regulated kinase (ERK) MAPK pathway that are also connected to cancer development [21]. In particular, mutations in *PTPN11* underlie two RASopathies, Noonan syndrome (NS) and NS with multiple lentigines (also called LEOPARD syndrome) [22,23]. SHP2 is also linked to insulin resistance based on its role downstream of the insulin receptor [24], mosaic somatic mutations that cause epilepsy [25], and loss of function *PTPN11* variants that cause metachondromatosis [26]. Due to the many important functions in disease contexts, SHP2 has received strong attention from both academia and the pharmaceutical industry.

Given the importance of correctly functioning SHP2, it needs to be tightly regulated. SHP2 contains three pY binding sites that are located in the catalytic PTP domain and in two SH2 domains, which are referred to as the N-SH2 domain (based on its *N*-terminal location in the protein) and the C-SH2 domain (situated between the N-SH2 domain and the PTP domain) [27]. The SH2 domains have multiple binding partners and interaction modes, with different outcomes depending on the specific pathway where SHP2 is acting [28]. In addition, SHP2 contains a flexible *C*-terminal tail that has several phosphorylation sites (PhosphoSitePlus database; phosphosite.org), of which pY542 and pY580 are the best studied [29–31]. In recent years, other post-translational modifications (PTMs) have been described, as well as non-catalytic functions and further mechanisms of SHP2 regulation. Furthermore, like other PTPs, SHP2 is redox-regulated through reactive oxygen species [32,33]. Here, we focus on the regulatory mechanisms of wildtype SHP2, summarizing first the more established roles of the SH2 domains and the pYs in the *C*-terminal tail, including recent insights, followed by describing a selection of new developments concerning non-catalytic functions as well as the mechanistic and regulatory understanding of SHP2. We refer the reader to other recent reviews for detailed insights into SHP2 in diseases [3,4,11,24], its redox regulation [32,33], and SHP2 drug discovery [3,4,11,27,34].

## Roles of the SH2 domains and the pYs in the *C*-terminal tail

**Figure 1:**
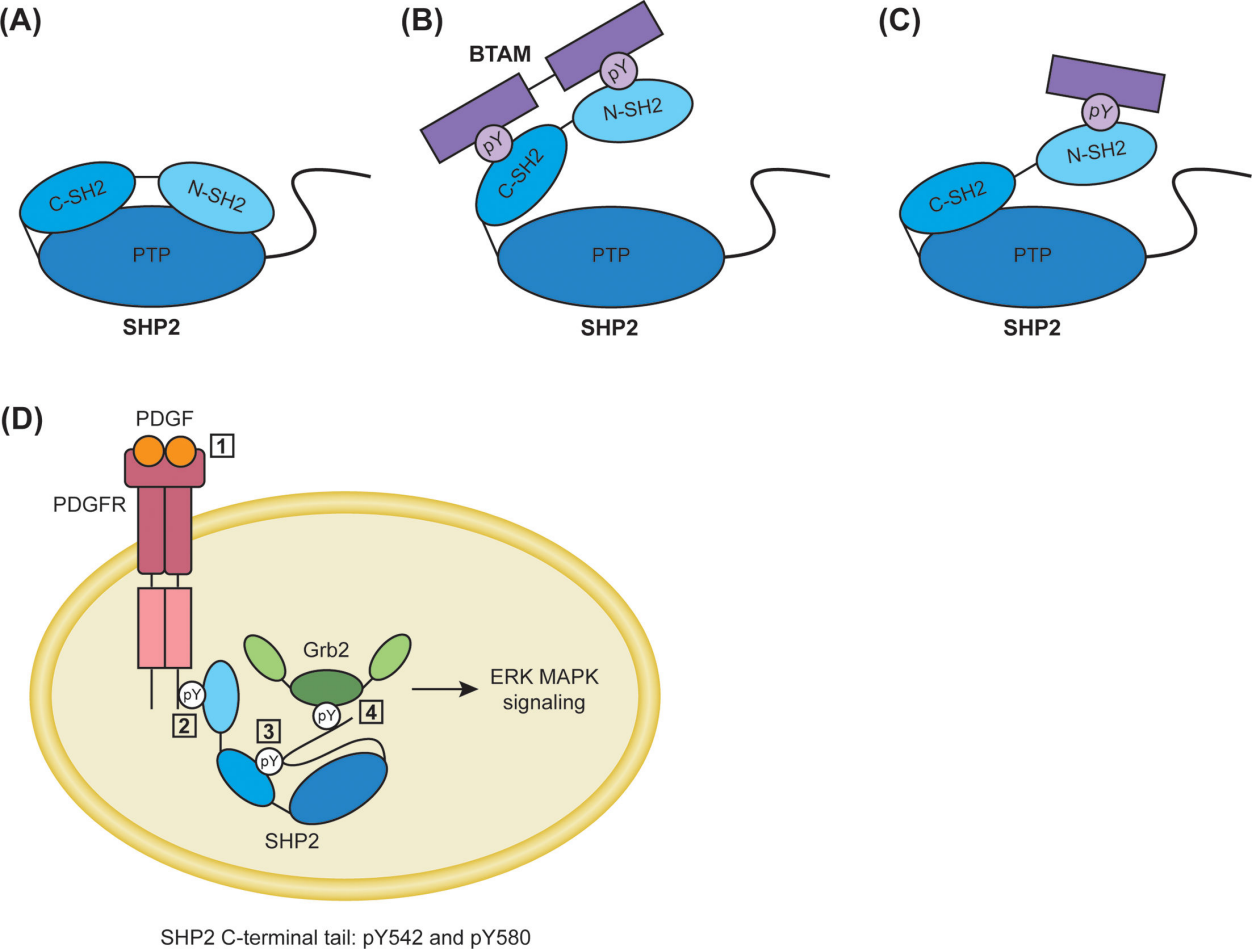
Roles of the SH2 domains and *C*-terminal tail pYs in SHP2 activation, catalytic activity, scaffolding, and signaling. (**A**) In the basal state, SHP2 resides in a closed autoinhibited conformation where the N-SH2 domain occludes the active site of the PTP domain. (**B**) Upon the binding of a bisphosphoryl tyrosine-based activation motif (BTAM) to both the N-SH2 and C-SH2 domains, this triggers a conformational change that results in a stable open conformation of SHP2, exposing the active site and fully activating the enzyme. (**C**) Activation of SHP2 can also occur by a single pY-motif that binds to the N-SH2 domain, which results in lower SHP2 activation compared with the binding of a BTAM. (**D**) Ligand binding of PDGF to its receptor PDGFR (*1*) results in PDGFR autophosphorylation on its cytoplasmic tail, which recruits SHP2 via its N-SH2 domain *(2*). This then promotes phosphorylation of the SHP2 *C*-terminal tail at Y542, which interacts with the C-SH2 domain (*3*) and facilitates phosphorylation of the SHP2 *C*-terminal tail at Y580. This subsequently recruits Grb2 (*4*) to trigger downstream ERK MAPK signaling. BTAM, bisphosphoryl tyrosine-based activation motif; pY, phosphotyrosine; PDGF, platelet-derived growth factor; PDGFR, platelet-derived growth factor receptor; Grb2, growth factor receptor-bound protein 2.

In its basal state, SHP2 resides in a closed autoinhibited conformation, in which the N-SH2 domain interacts with the PTP domain (Figure 1A). This interaction obstructs the catalytic pocket of the PTP domain and, simultaneously, distorts the pY-peptide-binding pocket of the N-SH2 domain [35], thus inhibiting the catalytic activity and reducing the pY-binding capability of the N-SH2 domain. The C-SH2 domain is not directly involved in the inhibitory function in the basal state and is unable to activate SHP2 on its own [36]. Instead, it is available to interrogate pY-binding motifs and for initial interactions with binding partners [37]. Proteins that carry two adjacent pY-containing motifs (also called a bisphosphoryl tyrosine-based activation motif, BTAM) [38], enable binding to the N-SH2 and C-SH2 domains simultaneously. Upon binding of the C-SH2 domain ligand, as part of a BTAM, the resulting increase in the local concentration of the N-SH2 domain ligand can counteract the inhibition of the N-SH2 domain by the PTP domain, allowing the N-SH2 to disengage and activating the enzyme [28] (Figure 1B). However, activation of SHP2 can also be accomplished by a single pY-motif-containing protein that binds to the N-SH2 domain [39] (Figure 1C). The binding of a BTAM leads to a stable open conformation of SHP2, exposing the active site and fully activating SHP2, compared with a lesser activation through N-SH2 binding alone [8,38,40,41]. In the SHP2 open conformation, the N-SH2 domain is located on a surface of the PTP domain opposite the active site, and the C-SH2 domain is rotated around 120° relative to the autoinhibited conformation [42], similar to the SHP1 open conformation where the C-SH2 domain also acts as a pivot to relocate the N-SH2 domain [43].

An interesting example of related proteins that can bind with either one or two pY-motifs to the SH2 domains of SHP2 is cytotoxin-associated gene A (CagA) effector proteins from *Helicobacter pylori*, a carcinogenic bacterium [39]. Upon delivery into gastric epithelial cells, CagA is phosphorylated at the Glu-Pro-Ile-Tyr-Ala (EPIYA) motif and binds to SHP2. This motif, and how often it repeats in CagA, varies in different *H. pylori* strains, with some containing only one motif. The surrounding amino acids, as well as the number of motifs, determine how strongly CagA interacts with SHP2 and how much SHP2 is activated. This can thereby modulate the extent of SHP2 deregulation that promotes neoplastic transformation in cells [39].

The interchange between mono- and bisphosphorylated ligand binding can be a means to regulate SHP2 activity. In the case of PD-1, full activation of SHP2 results from the binding of the phosphorylated immunoreceptor tyrosine-based inhibitory motif (ITIM) to the N-SH2 domain and the phosphorylated immunoreceptor tyrosine-based switch motif (ITSM) to the C-SH2 domain, locking SHP2 in the stable open conformation [8]. If ITIM is not phosphorylated or phosphorylated ITSM is present at high concentrations (highly up-regulated, dimerized PD-1), two ITSMs of two PD-1 proteins can bind to both SH2 domains of one SHP2 protein. This is caused by the stronger binding affinity of ITSM to both SH2 domains compared with ITIM and leads*—in vitro*—to dampened SHP2 activity by destabilizing its open conformation but can still result in robust SHP2 activation when PD-1 is only mono-phosphorylated in cells [8,44]. In addition to the role of the N-SH2 domain in SHP2 activation, it also carries a mitochondria-targeting sequence at its very *N*-terminus, an RRWFH motif. Translocase of the outer and inner membrane complexes recognize this motif and mediate SHP2 translocation into the mitochondrial matrix upon NLRP3 inflammasome activation [20].

In the SHP2 *C*-terminal tail, Y542 and Y580 can be phosphorylated by the platelet-derived growth factor receptor (PDGFR) upon receptor stimulation by PDGF [31]. Other receptor agonists, such as fibroblast growth factor (FGF), but not all, for example, epidermal growth factor (EGF), can induce phosphorylation of these sites [45]. It was suggested that they function as a docking site for the growth factor receptor-bound protein 2 (Grb2)/Son of sevenless (Sos) complex to activate the ERK MAPK pathway [31], whereby SHP2 acts as an adaptor protein. Early studies aimed to dissect the contributions of pY542 and pY580 to a potential intramolecular (*cis*-) binding to the N-SH2 and C-SH2 domains and to determine whether this conformational change has an influence on enzymatic phosphatase activity. Non-hydrolyzable phosphonates as pY mimics were incorporated into wildtype SHP2 or the inactive C459S mutant through native chemical ligation [29,30]. However, recently it was shown that phosphonates instead of phosphates on tyrosine are poorly binding mimics for pY concerning the C-SH2 domain [36], and the C459S mutant was observed to be catalytically dead but not present in the closed autoinhibited conformation [46]. Therefore, the conclusions of these earlier studies need to be revisited. In an effort to shed light on the roles of these two *C*-terminal tail pY sites in cells, Sun et al. applied fluorescence resonance energy transfer reporter constructs that included the native pY [31]. These studies showed, in mouse embryonic fibroblasts, that pY580 is the docking site for Grb2, while pY542 binds in *cis* to the C-SH2 domain. The N-SH2 domain was not involved in intramolecular binding with either pY site. Accordingly, upon ligand binding, PDGFRβ undergoes autophosphorylation at Y763 and Y1009, which are docking sites for SHP2 binding to PDGFRβ [47,48] and lead to the recruitment of SHP2 through its N-SH2 domain. Then, SHP2 pY542 is preferentially phosphorylated and engages in the *cis*-interaction with the C-SH2 domain, thereby facilitating pY580 phosphorylation [31]. This, in turn, leads to the recruitment of Grb2/Sos to trigger ERK MAPK signaling and sustained ERK phosphorylation (Figure 1D). Thus, here pY542 is involved in *cis*-interactions with the C-SH2 domain, while pY580 functions as a docking site in this role of SHP2 as adaptor protein [31].

## Recently emerged catalytic versus non-catalytic and regulatory mechanisms of SHP2

### Non-catalytic versus catalytic effects of SHP2

**Figure 2:**
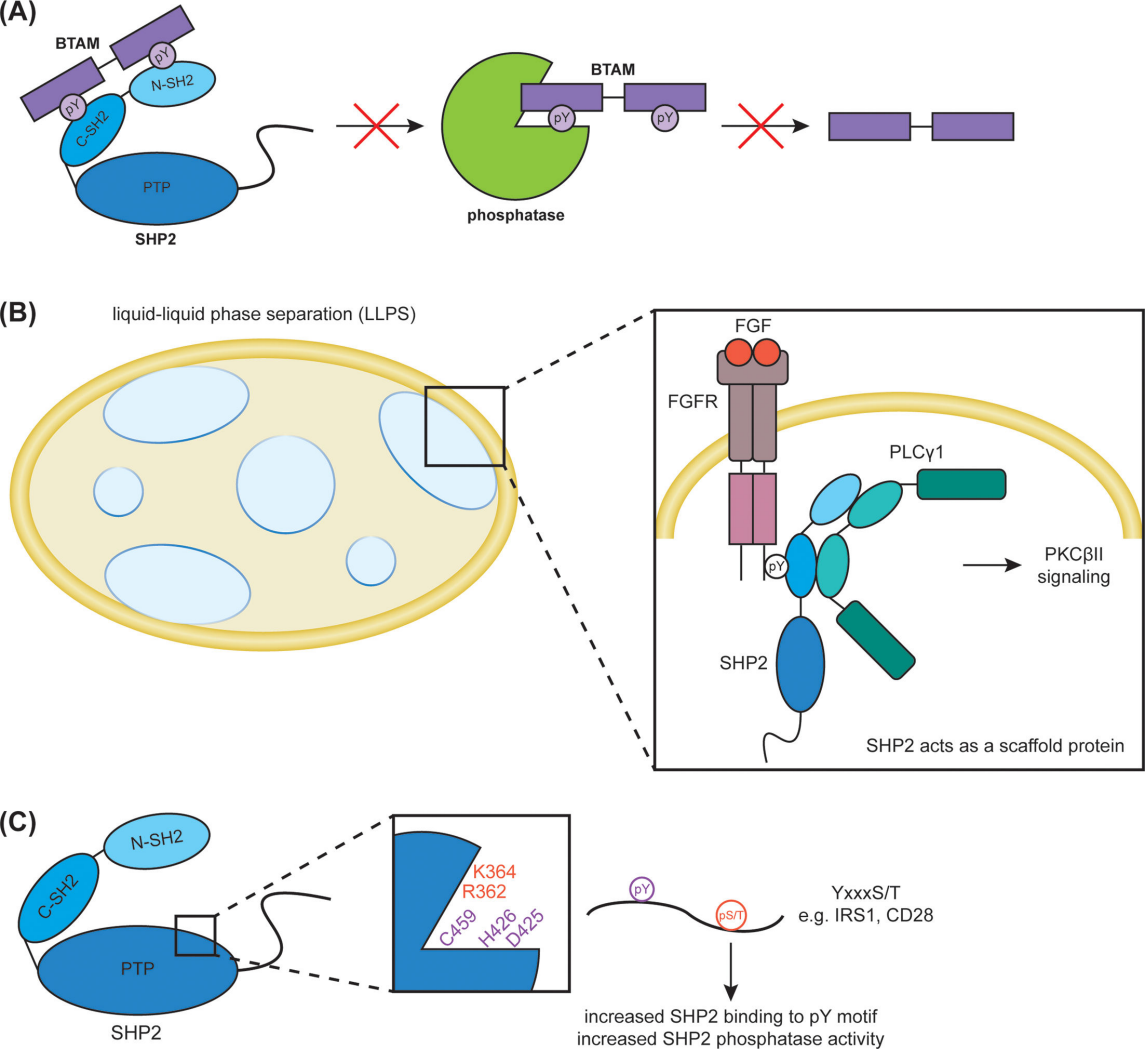
Novel insights into SHP2 function and regulation. (**A**) The tandem SH2 domains of SHP2 shield pY residues of protein binding partners from dephosphorylation. The binding of BTAM-containing proteins to the N-SH2 and C-SH2 domains of SHP2 protects the pYs from phosphatase-mediated dephosphorylation. (**B**) SHP2 complexes are involved in liquid–liquid phase separation (LLPS). Ligand binding of FGF to its receptor FGFR results in pY autophosphorylation in the cytoplasmic tail of FGFR, which recruits SHP2 via its C-SH2 domain. This promotes the interaction between SHP2 and PLCγ1 via the tandem SH2 domains of both proteins, without disrupting the SHP2 C-SH2 domain binding to the FGFR2 pY. This ternary complex, where SHP2 acts as an adaptor protein, drives downstream PKCβII signaling. (**C**) Phosphorylation of S/T proximal to the substrate pY regulates the activity of SHP2. SHP2 substrates, such as IRS1 and CD28, contain a pYxxxS/T sequence where the pS/T residue increases SHP2 binding to and dephosphorylation of the pY residue, mediated by R362 and K364 located in the SHP2 active site. LLPS, liquid–liquid phase separation; FGF, fibroblast growth factor; FGFR, fibroblast growth factor receptor; PKCβII, protein kinase C beta II; PLCγ1, phospholipase C gamma 1; pY, phosphotyrosine; S, serine; T, threonine; IRS1, insulin receptor substrate 1.

Recent phosphoproteomic approaches have delivered valuable insights into SHP2 signaling and substrates [49–51]. In one such study, a time-resolved phosphoproteomics readout was applied after EGF stimulation of epidermal growth factor receptor (EGFR)-elevated MDA-MB-468 cells, and allosteric SHP2 inhibition via treatment with the compound SHP099, which traps SHP2 in the autoinhibited closed conformation, was compared with non-inhibitor-treated cells [49]. Elevated tyrosine phosphorylation was found in early and late responders, which was interpreted as direct and potentially indirect or delayed direct dephosphorylation by SHP2. The phosphoproteomics data produced a wealth of potential new SHP2 substrates, of which three (Rho GTPase-activating protein 35 [ARHGAP35], phospholipase C gamma 2 [PLCγ2], and Occludin) were confirmed in follow-up studies. Importantly, reduced tyrosine phosphorylation upon SHP2 inhibition was also observed. A detailed investigation revealed a role for SHP2 in increasing the half-life of pY sites that are protected by binding of the two SHP2 SH2 domains, which is disrupted upon allosteric inhibition that traps SHP2 in the closed conformation [49] (Figure 2A). Indeed, this idea of SH2 domains protecting pY sites from dephosphorylation was first described in the 1990s by Timms et al. for SHP1 [52].

Another recent study identified a phenotype associated with the catalytically inactive yet conformationally open C459S mutant [53]. This variant impaired ciliary development, contributing to hydrocephalus in mice. The SHP2 E76K mutant, which also promotes the open conformation but has high catalytic activity, similarly leads to hydrocephalus development. Allosteric inhibition of SHP2, where both active and inactive variants are trapped in the closed conformation, alleviated the hydrocephalus phenotype. These effects suggest that SHP2-driven hydrocephalus arises primarily from conformational alterations rather than changes in enzymatic activity, supporting the importance of SHP2 as an adaptor protein and the potential of therapeutic intervention through allosteric inhibition [53]. It also shows that allosteric inhibition of SHP2 by trapping it in its closed conformation, which is a strong focus of current drug discovery efforts [34], cannot alone distinguish between adaptor and enzymatic functions. For this, inhibitors with different modes of action that selectively target the N-SH2 domain [54], C-SH2 domain [36], or active site [55] are valuable tools.

The recruitment of effector proteins to stimulated RTKs, which are phosphorylated on several sites, needs to be rapid and organized. To study whether this process could be supported by the preorganization of signaling proteins in discrete pools of high effective protein concentration rather than being reliant on probabilistic random diffusion, Lin et al. investigated the involvement of liquid–liquid phase separation (LLPS) [56]. Interacting proteins in membraneless droplets concentrate functional proteins at sites of action, enhancing equilibrium binding and enzyme activity [57]. Lin et al. reported that SHP2 formed LLP-separated states with several phosphorylated RTK cytoplasmic tails *in vitro*, which also included other signaling proteins such as PLCγ1. In cells, these complexes presented with liquid-like dynamic behavior at the plasma membrane upon stimulation with FGF. In the ternary complex composed of pFGFR2, SHP2, and PLCγ1, the interaction of pFGFR2 with SHP2 was mediated by the pY on the cytoplasmic tail of fibroblast growth factor receptor 2 (FGFR2) and the C-SH2 domain of SHP2. Remarkably, the interaction of PLCγ1 with SHP2 was concluded to result from dimerization of the tandem SH2 domains of both proteins, without interfering with the C-SH2 binding of SHP2 to pFGFR2. While LLPS appeared to be driven by the tandem SH2 domains of SHP2, it remained unclear whether LLPS drives the specific signaling complex formation and is thus required for function or if it is solely a consequence of the complex formation. SHP2 showed reduced phosphatase activity in this complex, likely enabling a sustained interaction with pFGFR2 and mediating PLCγ1/protein kinase C beta II (PKCβII) signaling [56] (Figure 2B). Thus, here SHP2 once again played the role of an adaptor/scaffold protein.

### Emerging roles of SHP2’s post-translational modifications (PTMs)

According to the database PhosphoSitePlus (phosphosite.org, data retrieved on June 12, 2025), different PTMs have been detected on SHP2, including phosphorylation on Y, serine (S) and threonine (T), acetylation on lysine (K), and ubiquitylation on K. The roles of most of these PTMs have not yet been studied. Of the phosphorylation sites, pY542 and pY580, both discussed above, as well as pY62, are most frequently detected. pY62 resides in the N-SH2 domain at the interface to the PTP domain and was reported to stabilize the open conformation of SHP2, thereby enhancing its catalytic activity [58]. It was found to be involved in the development of resistance to the allosteric inhibitor SHP099 in acute myeloid leukemia. In this context, SHP2 inhibition triggered a feedback loop leading to Fms-like tyrosine kinase 3 (FLT3) receptor activation, which in turn phosphorylated pY62, activating SHP2 and restoring ERK MAPK signaling [7].

In addition, SUMOylation on K590 in the *C*-terminal tail of SHP2 has been reported [59]. This modification can be reversibly removed by the small ubiquitin-like modifier (SUMO)1-specific protease SENP1. Functionally, SUMOylation promoted full ERK activation downstream of EGFR after stimulation with EGF. SHP2 is known to associate at the plasma membrane with EGFR and GRB2-associated-binding protein 1 (Gab1). While SHP2 SUMOylation did not itself have an effect on the catalytic activity, it instead mediated membrane recruitment. Gab1 has a SUMO-interaction motif [60], and the authors showed that SUMOylated SHP2 can be more effectively recruited to the plasma membrane than a SUMOylation-deficient mutant, likely enhancing the activation of the ERK MAPK pathway downstream of EGFR by supporting the recruitment of SHP2 to its localization in the cell where it acts [59].

### New means of SHP2 activation

Grb2 is known to bind to pY580 on SHP2, which is phosphorylated upon RTK stimulation, for recruitment to the plasma membrane [31] (Figure 1D). An alternative scenario was described that occurs under non-stimulated conditions. Non-phosphorylated Grb2 exists in cells in an equilibrium between dimer and monomer, a balance that can be tilted to one or the other state depending on intracellular conditions [61–64]. For example, when Grb2 is phosphorylated on Y160 by basal RTK activity, the monomeric state is prevalent [64]. In the absence of growth factors, this Y160-phosphorylated Grb2 was reported to be able to bind to SHP2 via a new bidentate interaction, where the PTP domain of SHP2 would bind to the Grb2 CSH3 domain and the SHP2 N-SH2 domain would interact with the Grb2 SH2 domain [65]. The interaction between the two SH2 domains appeared to be still intact when both were mutated to lose their ability to bind pY and seems therefore independent of Y-phosphorylation. The binding of the Grb2-SH2 to the SHP2-N-SH2 was reported to induce a conformational change that released SHP2 from its closed conformation and enhanced SHP2 phosphatase activity. Thus, this study concluded that SHP2 activation can also be triggered in the absence of growth factor stimulation or oncogenic mutations, providing insights into SHP2 behavior under basal conditions [65].

Furthermore, not only interacting proteins but also substrates can affect SHP2 phosphatase activity. The SHP2 substrates insulin receptor substrate 1 (IRS1) and CD28 carry a pYxxxS/T sequence, where the pY can be dephosphorylated by SHP2. Phosphorylation of the S/T residue increased, in both cases, the binding of SHP2 to the peptide sequences [66]. Sequence alignment of established SHP2 substrates indicated that SHP2 prefers a negative charge in the -4 and +4/5 positions respective to pY. The phosphorylation of S/T either in position +4 or -4 both increased SHP2 phosphatase activity on the substrate. Structural and computational investigations showed that R362 and K364 of SHP2 are responsible for this preference (Figure 2C) and that they also stabilize the open pre-catalytic state, which appears to be remarkably stable compared with other PTPs [66]. Thus, this study supports that reversible phosphorylation of an adjacent S/T to the substrate pY regulates the activity of SHP2.

## Conclusion

SHP2 regulation through its domains, PTMs, substrates, and interactions is remarkably complex, and the functions of SHP2 as an adaptor protein and active phosphatase enzyme complicate the understanding of its specific roles. While our knowledge in this area is increasing rapidly, there is still much to learn. For example, many PTMs are yet to be investigated, new means of regulation continue to emerge, and it is still largely a mystery how the complex interplay of SHP2 involvement in T cell activation downstream of the T cell receptor and in T cell inhibition downstream of PD-1 is regulated. Ultimately, these research efforts will inform new strategies for therapeutically targeting SHP2 in an effective and selective manner in the various diseases where SHP2 has been implicated.

**Perspectives**

- Src homology 2 domain-containing phosphatase-2 (SHP2) is a proto-oncogene, acts downstream of oncogenes, is frequently mutated in developmental disorders, is involved in immune cell signaling, and is an important drug target. Therefore, it is important to understand its molecular regulation and functions in order to enable specific therapeutic targeting in different disease contexts.
- SHP2 is regulated through multiple mechanisms such as those involving its SH2 domains, post-translational modifications (PTMs), and active site substrate recognition. It functions both as a phosphatase and as an adaptor/scaffold protein. Knowledge of these regulatory mechanisms and functions has been gathered for more than two decades. However, recently emerged studies provide important new insights into SHP2 regulation and activity in different cellular contexts.
- These new insights reveal the marvelous complexity of SHP2 regulation and function. Many questions still remain, concerning, for example, the complexity of SHP2 in immune cells and the roles of its different PTMs. Answers to these questions will inform new strategies for therapeutically targeting SHP2 in an effective and selective manner.

## Open Access

This article has been published open access under our Subscribe to Open programme, made possible through the support of our subscribing institutions, learn more here: https://portlandpress.com/pages/open_access_options_and_prices#conditional

## Abbreviations

ARHGAP35: Rho GTPase-activating protein 35
BTAM: bisphosphoryl tyrosine-based activation motif
CagA: cytotoxin-associated gene A
EGF: epidermal growth factor
EGFR: epidermal growth factor receptor
ERK: extracellular signal-regulated kinase
FGF: fibroblast growth factor
FGFR: fibroblast growth factor receptor
Gab1: GRB2-associated-binding protein 1
Grb2: growth factor receptor-bound protein 2
IRS1: insulin receptor substrate 1
ITIM: immunoreceptor tyrosine-based inhibitory motif
ITSM: immunoreceptor tyrosine-based switch motif
K: lysine
LLPS: liquid–liquid phase separation
MAPK: mitogen-activated protein kinase
NLRP3: NOD-like receptor family pyrin domain containing 3
NS: Noonan syndrome
PD-1: programmed cell death protein 1
PDGF: platelet-derived growth factor
PDGFR: platelet-derived growth factor receptor
PKCβII: protein kinase C beta II
PLCγ1/2: phospholipase C gamma 1/2
PTM: post-translational modification
PTP: protein tyrosine phosphatase
RAS: rat sarcoma virus
RTK: receptor tyrosine kinase
S: serine
SH2: Src homology 2
SHP2: Src homology 2 domain-containing phosphatase-2
SUMO: small ubiquitin-like modifier
Sos: Son of sevenless
T: threonine
TLR: Toll-like receptor
Y: tyrosine
pY: phosphorylated tyrosine
